# Automatic opportunistic osteoporosis screening in routine CT: improved prediction of patients with prevalent vertebral fractures compared to DXA

**DOI:** 10.1007/s00330-020-07655-2

**Published:** 2021-01-28

**Authors:** Maximilian T. Löffler, Alina Jacob, Andreas Scharr, Nico Sollmann, Egon Burian, Malek El Husseini, Anjany Sekuboyina, Giles Tetteh, Claus Zimmer, Jens Gempt, Thomas Baum, Jan S. Kirschke

**Affiliations:** 1 Department of Diagnostic and Interventional Neuroradiology, School of Medicine, Klinikum rechts der Isar, Technical University of Munich, Munich, Germany; 2 Department of Diagnostic and Interventional Radiology, University Medical Center Freiburg, Freiburg im Breisgau, Germany; 3 TUM-Neuroimaging Center, Klinikum rechts der Isar, Technical University of Munich, Munich, Germany; 4 Department of Diagnostic and Interventional Radiology, University Hospital Ulm, Ulm, Germany; 5 Department of Informatics, Technical University of Munich, Munich, Germany; 6 Department of Neurosurgery, School of Medicine, Klinikum rechts der Isar, Technical University of Munich, Munich, Germany

**Keywords:** Osteoporosis, Spine, Bone mineral density, Multidetector computed tomography, Neural networks

## Abstract

**Objectives:**

To compare spinal bone measures derived from automatic and manual assessment in routine CT with dual energy X-ray absorptiometry (DXA) in their association with prevalent osteoporotic vertebral fractures using our fully automated framework (https://anduin.bonescreen.de) to assess various bone measures in clinical CT.

**Methods:**

We included 192 patients (141 women, 51 men; age 70.2 ± 9.7 years) who had lumbar DXA and CT available (within 1 year). Automatic assessment of spinal bone measures in CT included segmentation of vertebrae using a convolutional neural network (CNN), reduction to the vertebral body, and extraction of bone mineral content (BMC), trabecular and integral volumetric bone mineral density (vBMD), and CT-based areal BMD (aBMD) using asynchronous calibration. Moreover, trabecular bone was manually sampled (manual vBMD).

**Results:**

A total of 148 patients (77%) had vertebral fractures and significantly lower values in all bone measures compared to patients without fractures (*p* ≤ 0.001). Except for BMC, all CT-based measures performed significantly better as predictors for vertebral fractures compared to DXA (e.g., AUC = 0.885 for trabecular vBMD and AUC = 0.86 for integral vBMD vs. AUC = 0.668 for DXA aBMD, respectively; both *p* < 0.001). Age- and sex-adjusted associations with fracture status were strongest for manual vBMD (OR = 7.3, [95%] CI 3.8–14.3) followed by automatically assessed trabecular vBMD (OR = 6.9, CI 3.5–13.4) and integral vBMD (OR = 4.3, CI 2.5–7.6). Diagnostic cutoffs of integral vBMD for osteoporosis (< 160 mg/cm^3^) or low bone mass (160 ≤ BMD < 190 mg/cm^3^) had sensitivity (84%/41%) and specificity (78%/95%) similar to trabecular vBMD.

**Conclusions:**

Fully automatic osteoporosis screening in routine CT of the spine is feasible. CT-based measures can better identify individuals with reduced bone mass who suffered from vertebral fractures than DXA.

**Key Points:**

• *Opportunistic osteoporosis screening of spinal bone measures derived from clinical routine CT is feasible in a fully automatic fashion using a deep learning-driven framework (**https://anduin.bonescreen.de**).*

• *Manually sampled volumetric BMD (vBMD) and automatically assessed trabecular and integral vBMD were the best predictors for prevalent vertebral fractures.*

• *Except for bone mineral content, all CT-based bone measures performed significantly better than DXA-based measures.*

• *We introduce diagnostic thresholds of integral vBMD for osteoporosis (< 160 mg/cm*^*3*^*) and low bone mass (160 ≤ BMD < 190 mg/cm*^*3*^*) with almost equal sensitivity and specificity compared to conventional thresholds of quantitative CT as proposed by the American College of Radiology (osteoporosis < 80 mg/cm*^*3*^*).*

**Supplementary Information:**

The online version contains supplementary material available at 10.1007/s00330-020-07655-2.

## Introduction

Osteoporosis is a metabolic bone disease characterized by impaired bone strength, predisposing the individual to an increased risk of fracture [1]. Osteoporosis affects the population worldwide, particularly the elderly in developed countries [2]. In the European Union, the economic burden of osteoporotic fractures has been estimated at 37 billion euros per year and is expected to increase by 25% in 2025 [3].

Besides hip fractures, vertebral fractures are the most common and most consequential osteoporotic fractures [4]. Their prevalence among Europeans older than 50 years ranges between 18 and 26% [5]. Vertebral fractures have dramatic consequences that include a reduced quality of life [6], a 2-fold increase in age-adjusted mortality risk [7], and a 3-fold increase in the risk of additional fractures compared to the normal population, respectively [8]. All types of osteoporotic fractures in the elderly foreshadow a high risk of poor outcomes, so that early medical intervention is strongly advised [9]. Medical treatment can specifically target patients with a very high risk profile and long-term management is generally required [10].

The main problem of osteoporosis is that osteoporotic patients remain asymptomatic until a fracture occurs. Moreover, osteoporotic vertebral fractures remain clinically silent with only 15–30% coming to clinical attention [11]. Thus, the primary aim in osteoporosis care is to identify people at high risk of fractures in order to initiate medical treatment before the first fracture occurs. To date, the standard screening method includes assessing clinical risk factors and measuring areal bone mineral density (aBMD) using dual-energy X-ray absorptiometry (DXA) [1]. However, there are two major concerns with this approach. First, less than half of women (44%) and even fewer men (21%) with osteoporotic fractures exhibited low aBMD in a large observational study [12], emphasizing the inherent inaccuracies of DXA [13]. Second, there is significant variability in the access to DXA services and many fall short of international quality standards [14]. Yet, other methods of bone densitometry exhibit even more disadvantages: quantitative computed tomography (QCT) has limited availability, is more expensive, and is associated with a substantially higher radiation dose (> 100-fold) [15]. Thus, an alternative method for osteoporosis screening that would be readily available and exhibits a higher accuracy than DXA in predicting major osteoporotic fractures is highly warranted.

With the advent of sufficient computational power “deep learning”, an approach to machine learning using layers of convolutional neural networks (CNNs), has lately become popular. Specifically, CNNs can increase efficiency and accuracy in segmentation tasks. We recently introduced a framework for fully automatic segmentation of vertebrae in any CT dataset within several seconds [16, 17]. This was a cornerstone for the implementation of an opportunistic screening tool that can extract spinal bone measures from any CT data in a fully automatic fashion. Opportunistic quantitative evaluation of preexisting clinical routing CT entails neither additional costs nor radiation exposure [15]. Building on this groundwork, we now aim to proof the concept of opportunistic osteoporosis screening using our fully automated framework (https://anduin.bonescreen.de) to assess various bone measures in clinical CT and to investigate their predictive value for vertebral fracture assessment.

The purpose of this study was to systematically compare the association between prevalent osteoporotic vertebral fractures and various measures of spinal bone mass, extracted from clinical routine CT both automatically and manually, with the reference standard of DXA.

## Methods

### Study population

The local institutional review board approved this monocentric retrospective study (ethics committee’s reference number 27/19S/SR) and waived written informed consent. In a query on all patients registered until May 2017 in the institutional database, we identified 360 patients who had DXA and CT available including parts of the thoracolumbar spine. The maximum interval between DXA and CT exams was defined as 12 months. We excluded patients with a history of vertebral metastasis or hematologic disorders (*n* = 18), without assessable lumbar DXA (*n* = 34), without assessable CT (due to visualization of fractured vertebrae only, tube voltage other than 120 kV, or severely limited image quality; *n* = 15), and patients younger than 50 years at the time of DXA examination (*n* = 35). CT scans of the remaining 258 patients were screened for prevalent osteoporotic vertebral fractures using the semi-quantitative technique by Genant [18]. Based on visual image review, patients were categorized either as fractured (if grade ≥ 1) or non-fractured. To enable a correct fracture classification and not miss a fracture that was not visualized due to partial coverage of the spine in the CT scan, non-fractured patients were excluded from the study if not at least vertebral levels T7 to L4 were visualized (*n* = 66). This yielded a final study group of 192 patients, with 148 patients (77%) showing at least one prevalent osteoporotic vertebral fracture.

### CT image acquisition

CT scans were performed on six different multidetector CT scanners (Philips Brilliance 64, iCT 256, and IQon, Philips Medical Systems; Siemens Somatom Definition AS, Somatom Definition AS+, and Somatom Sensation Cardiac 64, Siemens Healthineers); some scans were performed after administration of either both oral (Barilux Scan, Sanochemia Diagnostics) and intravenous (Iomeron 400, Bracco) contrast medium or only intravenous contrast material (*n* = 61). Image data were acquired with all scanners in helical mode with a peak tube voltage of 120 kVp, a slice thickness of 0.9–1 mm, and adaptive tube load. Post-contrast scans were acquired in either the arterial or portal venous phase, triggered by a threshold of CT attenuation surpassed in a region of interest placed in the aorta or after a delay of 70 s, respectively, depending on the clinical indication for CT imaging. Sagittal reformations of the spine with 1-, 2-, or 3-mm slice thickness were reconstructed with a bone kernel and used for further analysis in this study. Imaging was performed for various indications not related to bone densitometry: acute back pain or suspected spinal fracture (*n* = 86); cancer staging, restaging, or follow-up (*n* = 55); exclusion of acute abdominal pathology (*n* = 21); chronic back pain (*n* = 14); and postoperative examination (*n* = 16).

### Dual-energy X-ray absorptiometry

Areal BMD of lumbar vertebrae L1 to L4 was assessed in anterior-posterior projection on a DXA scanner (GE Lunar Prodigy, GE Healthcare). Scans were performed by trained technologists and quality was assured through evaluation by experienced physicians following current recommendations [19]. Those skeletal sites affected by severe local structural changes or artifacts were excluded. T-Scores were calculated in relation to a reference population of healthy young women who are at their peak bone mass. The overall lowest T-score at the lumbar spine was reported and accounted for the diagnosis of osteoporosis [20]. Osteoporosis was defined as T ≤ − 2.5 SD and low bone mass as − 2.5 < T ≤ − 1 SD [21].

### Opportunistic CT-based measurements of bone mass

Volumetric and areal measures of bone mass were extracted from clinical CT scans in at least one of vertebrae T12 to L4. Measurements were averaged in case multiple levels could be evaluated.

#### Asynchronous calibration and correction for contrast medium

CT attenuation in Hounsfield units (HU) was converted to volumetric BMD using asynchronous calibration. In asynchronous calibration, phantoms with elements of bone-equivalent density are scanned to calculate HU-to-BMD relations that are specific for a certain CT scanner and acquisition protocol. Previously published HU-to-BMD conversion equations were used for all CT scanners in this study [22]. Most of these conversion equations were established in scans of a phantom with hydroxyl-apatite inserts of known density in milligrams per cubic centimeter (Anthropomorphic Abdomen Phantom, QRM Quality Assurance in Radiology and Medicine). Bias of BMD values due to intravenous injection of contrast medium was corrected for using linear correction equations for arterial and portal/venous contrast phases [23]. HU values were converted to BMD and corrected for the presence of contrast medium prior to any subsequent evaluation of CT data.

#### Automatic extraction of volumetric bone measures

Volumetric measures were extracted in an automatic multi-step procedure, which required minimal user interaction and was implemented in Python. First, vertebrae were automatically segmented in CT scans using a framework of CNNs that identifies the spine, labels each vertebral body, and creates segmentation masks [16]. Second, vertebral bodies were separated from posterior elements in these masks using affine and deformable transformations to fit templates of vertebral subregions to each vertebral level. Third, segmentation masks of vertebral bodies were used to extract integral vBMD and bone mineral content (BMC) or additionally eroded by 5 mm to exclude cortical bone for sampling trabecular vBMD.

#### CT-Based areal BMD

**Fig. 1:**
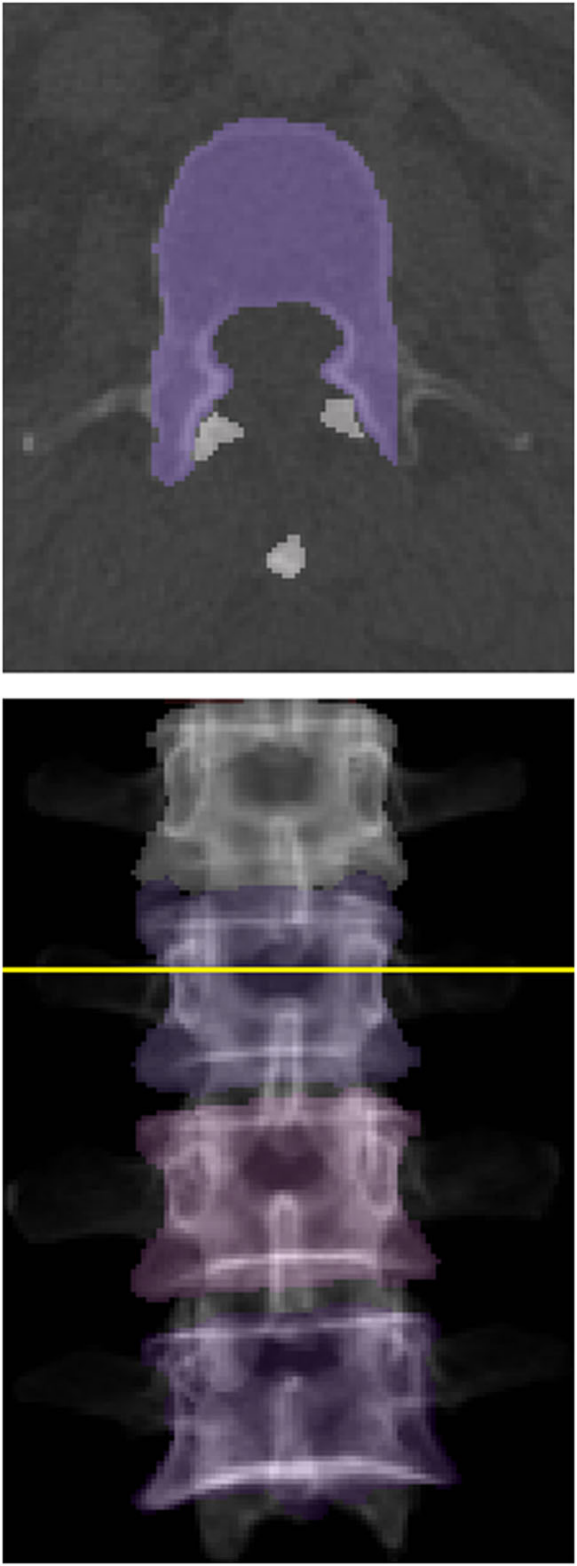
Contour of vertebra L2 in axial cut (top) used for generation of CT-based DXA of L1 to L4 in virtual posterior-anterior projection (bottom)

Areal BMD was extracted from virtual DXA-equivalent scans created from CT data (CT-based aBMD) for vertebrae L1 to L4. Only bony tissue inside the vertebral segmentation masks was included in the virtual images created in posterior-anterior projection. We chose this approach to take advantage of the 3-dimensional character of CT scans compared to DXA, thus postulating its superior accuracy notwithstanding the fact that it is a monoenergetic technique. Areal BMD was sampled from the posterior-anterior projections in overlay masks corresponding to the contour of vertebral bodies, thus excluding lateral processes (Fig. 1). Good correlation between CT-based and DXA-based aBMD of L2 and L3 (*R*^2^ = 0.814 and *R*^2^ = 0.739, respectively) could be shown for a sample group of 29 patients (22 women, mean age 61.5 ± 13.6 years; Suppl. Fig. 1). Bland-Altman plots showed a bias of − 0.054 and − 0.015 g/cm^2^ at L2 and L3, respectively, for CT-based aBMD (Supplementary Fig. 1). Thus, CT-based assessment seemed to slightly underestimate aBMD compared to DXA.

#### Quality assurance in evaluation survey

**Fig. 2:**
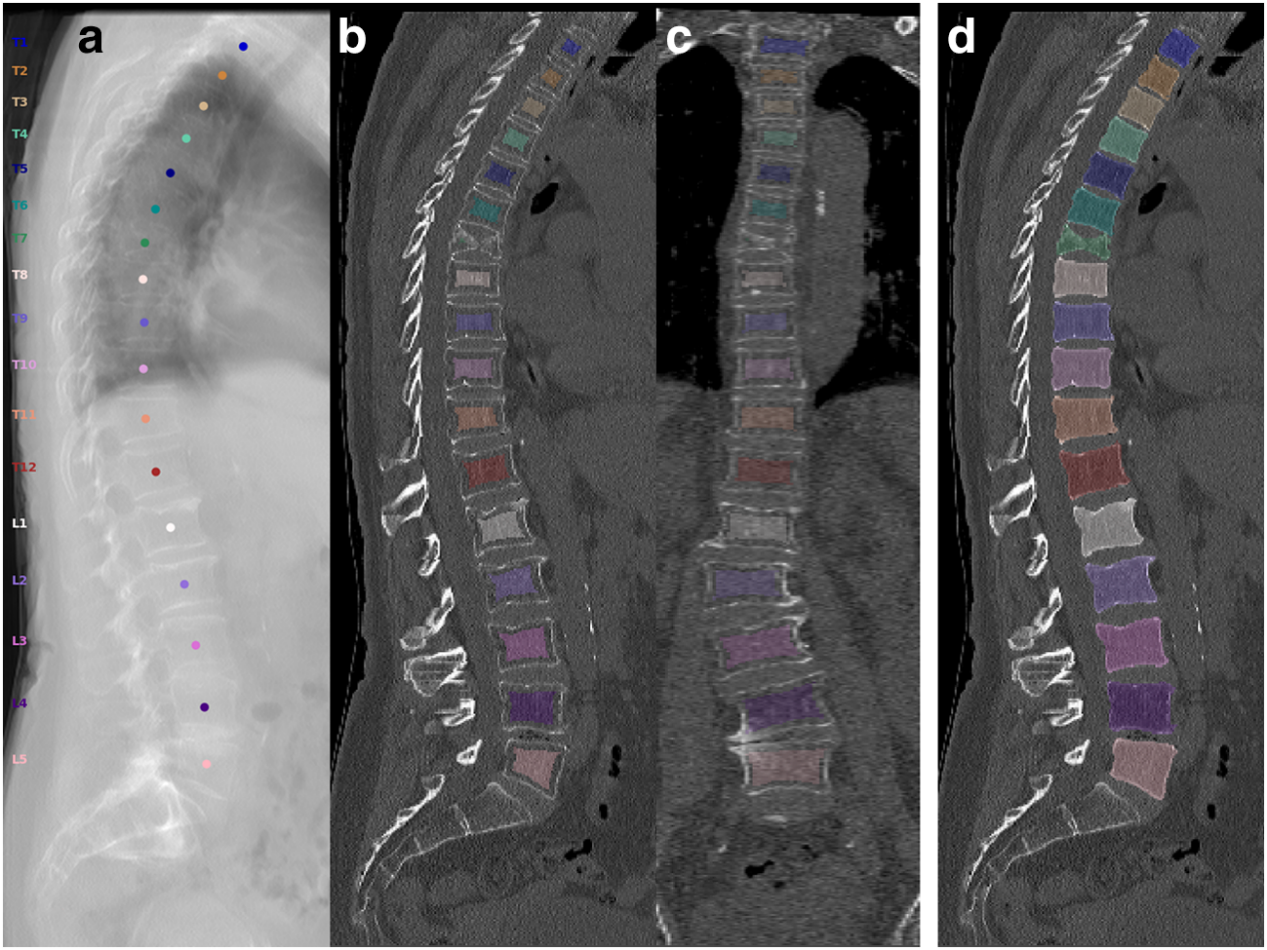
CT scan of an 80-year-old woman with acute back pain visualized as virtual radiograph in lateral projection (**a**) and curved planar reconstructions in lateral and coronal views (**b**, **c**, **d**). A severe crush fractures is visualized at T7 besides multiple mild to moderate vertebral deformities. Mild scoliotic deformity at the thoracolumbar junction and spondylosis with sclerosis (equivalent to Modic III in MRI) is present at L4/5. Therefore, L4 was excluded from assessment. T12 to L3 yielded a mean trabecular vBMD of 26.6 mg/cm^3^, integral vBMD of 135.4 mg/cm^3^, CT-based aBMD of 0.768 g/cm^2^, and BMC of 4.45 g. Trabecular and integral vBMD are clearly in the osteoporotic range. DXA reported T-score of − 2.5 SD still in the range of low bone mass (not shown). Masks for extraction of trabecular (**b**, **c**) as well as integral vBMD (**d**) are shown as colored overlays. Colored points in the virtual radiograph are automatically estimated by the labelling CNN and represent the vertebral body centroids. Lateral and coronal curved planes are reconstructed by interpolation through these points

**Fig. 3:**
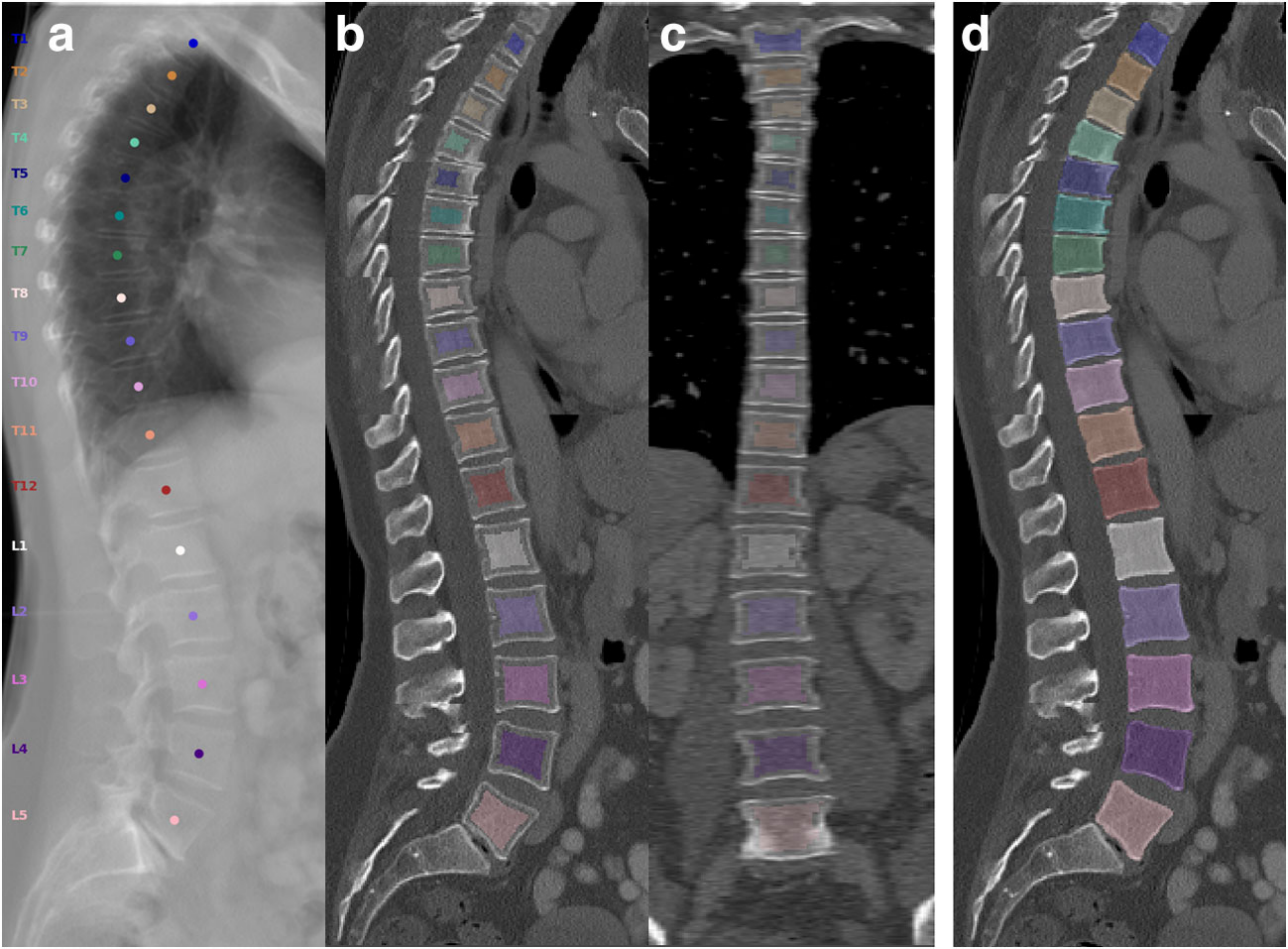
CT scan of a 50-year-old woman performed for breast cancer staging visualized as virtual radiograph in lateral projection (**a**) and curved planar reconstructions in lateral and coronal views (**b**, **c**, **d**). No osteoporotic vertebral fracture is visualized. There are signs of spondylosis at L5/S1. Assessment of T12 to L4 yielded a mean trabecular vBMD of 134.7 mg/cm^3^, integral vBMD of 204.5 mg/cm^3^, CT-based aBMD of 1.008 g/cm^2^, and BMC of 6.18 g. DXA reported T-score of − 2.2 SD (not shown). Trabecular and integral vBMD concur with normal bone mass. DXA T-score corresponds to low bone mass with tendency towards the threshold for osteoporosis (< − 2.5 SD). Masks for extraction of trabecular (**b**, **c**) as well as integral vBMD (**d**), are shown as colored overlays. For more details on image creation please refer to Fig. 2 and “Methods”

Curved planar reconstructions (CPRs) in sagittal and coronal view passing through the centroids of vertebral bodies were generated from CT data and overlaid with segmentation masks at 40% opacity. Additionally, virtual radiographs in lateral projection were calculated from CT data. These image reconstructions served as a survey to identify vertebral levels that had to be excluded from bone mass assessment due to (1) vertebral fractures, (2) degenerative changes, or (3) other abnormalities (e.g., foreign material) that led to alterations in bone mass not specific to osteoporosis (Figs. 2 and 3).

### Clinical thresholds for volumetric BMD measures

For trabecular vBMD, we used the diagnostic thresholds for osteoporosis (BMD < 80 mg/cm^3^) and for low bone mass (80 ≤ BMD ≤ 120 mg/cm^3^) proposed by the American College of Radiology (ACR) [24]. For integral vBMD, we developed new diagnostic thresholds in relation to the cut points for trabecular vBMD. Therefore, we compared the coordinate points in receiver operating characteristics (ROC) analysis between trabecular and integral vBMD and determined those points for integral vBMD with the smallest geometrical distance to the respective cut points of trabecular vBMD, thus yielding sensitivity and specificity that matched most closely for both measures. Cutoff values in milligrams per cubic centimeter were rounded to the nearest step of 5 mg/cm^3^.

### Statistical analysis

Study group characteristics were compared between patients with and without prevalent vertebral fractures using a two-sample *t* test for continuous variables and a chi-squared test of independence for sex. We investigated the association between different bone measures and prevalent fracture status in logistic regression, calculating odds ratios (ORs) and 95% confidence intervals (CIs) for one SD change. Models were additionally adjusted for age and sex. Area under the curve (AUC) was calculated in ROC analysis to test the classification performance of all bone measures to predict prevalent osteoporotic vertebral fractures. ROC curves were compared with DeLong’s test for two correlated ROC curves using the pROC package [25]. Statistical analyses were conducted using SPSS (version 26; IBM) and RStudio (version 1.3.1073; RStudio). Statistical significance was set at a level *p* < 0.05 for all statistical tests.

## Results

Overall, 192 patients (141 women, 51 men) with a mean age of 70.2 ± 9.7 years were included in this study. Fractured patients (107 women, 41 men; Fig. 2) were significantly older (72.0 ± 9.3 years vs. 64.3 ± 8.6 years, *p* < 0.001) and showed significantly lower values in all bone measures (DXA-based aBMD, CT-based aBMD, manual vBMD, trabecular vBMD, integral vBMD, and BMC, *p* < 0.001 each; DXA-based T-score, *p* = 0.001) compared to patients without fractures (Table 1; Fig. 3).

**Table 1:** Study group characteristics stratified by fracture prevalence

Variable	No fracture (*n* = 44)	Fracture (*n* = 148)	No fracture vs. fracture	Total (*n* = 192)
Women, *n* (%)	34 (77%)	107 (72%)	n.s.	141 (73%)
Age, years, mean (SD)	64.3 (8.6)	*72.0 (9.3)*	*p* < 0.001	70.2 (9.7)
DXA-based T-score, mean (SD)	− *1.4 (1.6)*	− 2.4 (1.6)	*p* = 0.001	− 2.1 (1.6)
DXA-based aBMD, g/cm^2^, mean (SD)	*1.076 (0.219)*	0.948 (0.204)	*p* < 0.001	0.978 (0.214)
CT-based aBMD, g/cm^2^, mean (SD)	*0.951 (0.204)*	0.752 (0.199)	*p* < 0.001	0.797 (0.217)
Manual vBMD, mg/cm^3^, mean (SD)	*119.7 (38.1)*	58.4 (32.7)	*p* < 0.001	72.4 (42.6)
Trabecular vBMD, mg/cm^3^, mean (SD)	*113.5 (34.3)*	62.8 (27.5)	*p* < 0.001	74.4 (36.1)
Integral vBMD, mg/cm^3^, mean (SD)	*188.0 (35.5)*	140.2 (32.1)	*p* < 0.001	151.2 (38.5)
CT-based BMC, g, mean (SD)	*6.42 (1.87)*	5.00 (1.68)	*p* < 0.001	5.33 (1.82)

Statistically significant values are in italics; *n.s.*, non-significant at *p* < 0.05

Prevalent vertebral fractures were significantly associated with all DXA- and CT-based bone measures irrespective of adjustment for age and sex (Table 2). However, there were considerable differences with stronger associations for all CT-based measures (ranging from OR = 2.5, 95% CI 1.7–3.9 for adjusted CT-based aBMD to OR = 7.3, 95% CI 3.8–14.3 for adjusted manual vBMD) compared to DXA-based measures (OR = 1.9, 95% CI 1.3–2.8 each for adjusted DXA-based T-score and aBMD) and for both adjusted and unadjusted ORs (Table 2).

**Table 2:** Association of prevalent vertebral fractures with normalized DXA- and CT-based bone measures calculated as odds ratio (*OR*) with 95% confidence interval (*CI*)

Factor	Odds ratio (95% CI)	
	Unadjusted	Adjusted for age and sex
DXA-Based T-score	1.8 (1.3–2.6)	1.9 (1.3–2.8)
DXA-Based aBMD	1.8 (1.3–2.6)	1.9 (1.3–2.8)
CT-Based aBMD	2.7 (1.8–4.0)	2.5 (1.7–3.9)
CT-Based BMC	2.1 (1.5–3.1)	3.0 (1.9–4.8)
Integral vBMD	4.8 (2.8–8.1)	4.3 (2.5–7.6)
Trabecular vBMD	6.8 (3.7–12.4)	6.9 (3.5–13.4)
Manual vBMD	7.7 (4.1–14.5)	7.3 (3.8–14.3)

**Fig. 4:**
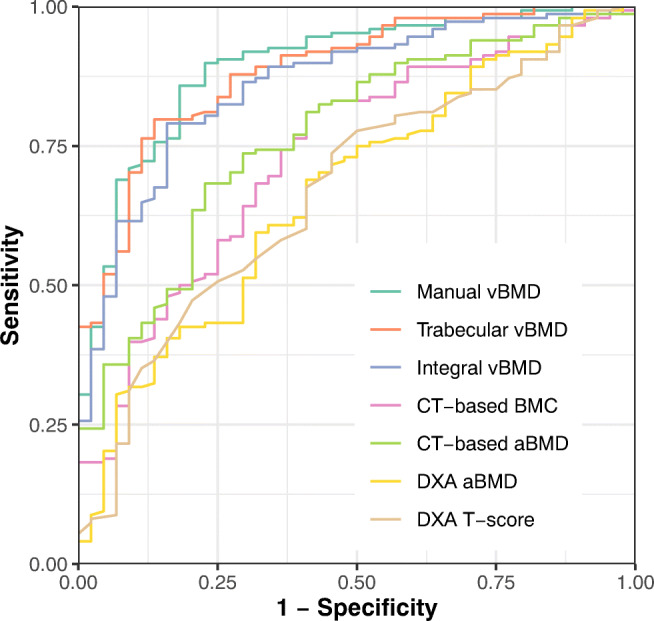
ROC plots for DXA- and CT-based bone measures used to classify fracture status of patients

AUC analysis showed that all bone measures were statistically significant predictors of prevalent vertebral fractures (Table 3, Fig. 4). However, most CT-based measures performed significantly better than DXA, e.g., when comparing DXA-based aBMD with intergral, trabecual, or manual vBMD (AUC = 0.668 vs. 0.735, 0.860, or 0.885, respectively, *p* < 0.001 each; Table 3). Only CT-based BMC showed no significant difference in discriminatory power to DXA-based aBMD or T-score (AUC = 0.735 vs. 0.668 or 0.67, respectively).

**Table 3:** Area under the ROC curve (AUC) for DXA- and CT-based bone measures classifying fracture status of patients

Classifier	AUC (95% CI)	Vs. DXA aBMD*	Vs. CT-based aBMD*
DXA-Based T-score	0.67 (0.581–0.759)	n.s.	*p* = 0.003
DXA-Based aBMD	0.668 (0.579–0.756)	-	*p* = 0.002
CT-Based aBMD	0.769 (0.693–0.845)	p = 0.002	-
CT-Based BMC	0.735 (0.653–0.818)	n.s.	n.s.
Integral vBMD	0.86 (0.801–0.92)	*p* < 0.001	*p* < 0.001
Trabecular vBMD	0.885 (0.833–0.938)	*p* < 0.001	*p* < 0.001
Manual vBMD	0.894 (0.841–0.947)	*p* < 0.001	*p* = 0.003

*p values for comparison of the respective AUC against the AUC of DXA-/CT-based aBMD; *n.s.* non-significant at *p* < 0.05

Diagnostic thresholds were determined for integral vBMD that define osteoporosis with BMD < 160 mg/cm^3^ and low bone mass with 160 ≤ BMD < 190 mg/cm^3^. Those cut points had almost equal sensitivity and specificity to predict patients with prevalent vertebral fractures compared to trabecular vBMD (84% vs. 86% sensitivity and 78% vs. 78% specificity for the osteoporosis threshold as well as 41% vs. 41% sensitivity and 95% vs. 98% specificity for the low bone mass threshold, respectively).

## Discussion

All automatically assessed CT-based bone measures had a highly significant association with the prevalence of osteoporotic vertebral fractures with no significant differences between automatic and manual measurements. Except for BMC, all CT-based bone measures showed significantly better discriminatory power for the prevalence of vertebral fractures compared to DXA-based measures.

We reported on elderly patients that all received DXA scans, thereby implicating that osteoporosis was already suspected; thus, the high prevalence of at least mild (Genant grade 1) osteoporotic vertebral fractures (77%) is not surprising. These differences in the study population—paired with the focus on prevalent instead of incident fractures and better sensitivity to detect fractures by using ≤ 1-mm instead of 2.5-mm axial slices [26]—could lead to the relatively higher ORs compared to Allaire et al (OR = 2.7 vs. 1.6 for CT-based aBMD, OR = 4.8 vs. 2.8 for integral vBMD, and OR = 6.8 vs. 2.1 for trabecular vBMD) [27]; though, CT-based BMC posed an exception (OR = 2.1 vs. 3.3). Similarly, the AUCs were comparatively higher than in the cited study (AUC = 0.769 vs. 0.715 for CT-based aBMD and AUC = 0.86 vs. 0.815 for integral vBMD); again, this is with the exception of CT-based BMC (AUC = 0.735 vs. 0.794). Of note, BMC is the only non-density measure of bone mass considered in this study. Therefore, the error introduced by bone mass without structural support for the vertebra’s compressive strength (e.g., spondylophytes) is not alleviated in any way.

Compared to a study of incident vertebral fractures in the Osteoporotic Fractures in Men (MrOS) Study cohort of elderly men, the AUCs we report seem considerably higher for trabecular vBMD (AUC = 0.885 vs. 0.79), but lower for DXA-based aBMD (AUC = 0.668 vs. 0.72) [28]. In another study on clinically identified vertebral fractures in the MrOS cohort, these results were paralleled with relatively higher values for integral vBMD (AUC = 0.86 vs. 0.82) and lower values for DXA-based aBMD (AUC = 0.668 vs. 0.76) [29]. In contrast to a community-dwelling population like the MrOS cohort, our study group has a selection bias of elderly hospital inpatients, mainly neurosurgical and oncological and exhibiting severe spinal degeneration that render areal density measures inaccurate [30]. In this context, BMC may become even more inaccurate, as outlined before.

Looking 25 years back in time, the insight that trabecular vBMD (QCT; AUC = 0.81) offers better discriminatory power for the prevalence of vertebral fractures than aBMD (DXA; AUC = 0.65) appears familiar [31]. Here, we were able to reproduce these results on modern scanner hardware. In this regard, a recently presented approach to directly estimate aBMD from CT scans using CNNs trained on DXA and CT data is questionable because it propagates the inaccuracies of DXA to CT measures [32]. Previously, efforts to automatically assess BMD in CT data have been undertaken [33]. Some automatic tools use HU as a proxy for BMD [34], which is a method that is expected to produce inaccuracies due to its lack of scanner-specific calibration to bone [35] as well as high variations due to presence of contrast material [15, 36, 37]. Of note, automatic assessment of other CT-derived biomarkers such as muscle attenuation has shown potential to predict fragility fractures [38]. In contrast to these previous studies, we report on calibrated bone measures (aBMD, vBMD, or BMC) that were fully automatically extracted using fast and reliable CNNs. Using an earlier version of this automatic framework we were able to predict screw loosening after lumbar spinal instrumentation in patients with osteoporotic trabecular vBMD [40]. Given that integral vBMD performed almost as good as trabecular vBMD, it would be convenient to have diagnostic thresholds available for integral vBMD that define osteoporosis and low bone mass similar to those defined by the ACR for trabecular vBMD [24]—an idea that has been previously proposed [27]. Here, we developed thresholds of integral vBMD for osteoporosis and for low bone mass. These diagnostic thresholds should be validated in follow-up studies investigating fracture risk because we did not report on incident vertebral fractures.

There are limitations to this retrospective study. As mentioned before, there was a selection bias of elderly and mainly neurosurgical and oncological patients because it was required that they had received both multidetector CT and DXA within 1 year. Thus, osteoporosis was already suspected. However, this is exactly the population that could benefit from opportunistic osteoporosis screening because CT scans already exist and DXA scans become prone to inaccuracies due to spinal degeneration. Moreover, oncological patients would particularly benefit from opportunistic screening because osteoporosis may occur as a side effect of cancer treatment [39].

In conclusion, this study showed that opportunistic and fully automatic assessment of areal and volumetric bone measures in clinical routine CT scans is feasible. Volumetric and integral vBMD showed the best performance of these automatic measures to predict vertebral fractures. DXA-based and non-volumetric measures performed relatively worse. Finally, we propose newly developed diagnostic thresholds of integral vBMD for osteoporosis (< 160 mg/cm^3^) and low bone mass (160 ≤ BMD < 190 mg/cm^3^) that should be validated in upcoming studies.

## Supplementary Information

ESM 1 (DOCX 53 kb)

## Abbreviations

aBMD: Areal BMD
ACR: American College of Radiology
AUC: Area under the ROC curve
BMC: Bone mineral content
BMD: Bone mineral density
CI: Confidence interval
CNN: Convolutional neural network
CPR: Curved planar reconstruction
DXA: Dual X-ray absorptiometry
HU: Hounsfield units
MDCT: Multidetector computed tomography
MRI: Magnetic resonance imaging
MrOS: Osteoporotic Fractures in Men Study
OR: Odds ratio
QCT: Quantitative computed tomography
ROC: Receiver operating characteristics
vBMD: Volumetric BMD
