# Supplementary material for: Automatic opportunistic osteoporosis screening in routine CT: improved prediction of patients with prevalent vertebral fractures compared to DXA
Source: Eur Radiol. 2021 Jan 28;31(8):6069–77. doi: 10.1007/s00330-020-07655-2 (PMC8270840; doi:10.1007/s00330-020-07655-2)

Supplemental Fig. 1: Linear regression of DXA- and CT-based areal bone mineral density for L2 and L3 (top) and corresponding Bland-Altman plot with 95% confidence intervals(dashed lines; bottom).

CT-based aBMD shows a negative bias of -0.054 g/cm² compared to DXA-based aBMD at L2 and of -0.015 g/cm² at L3.


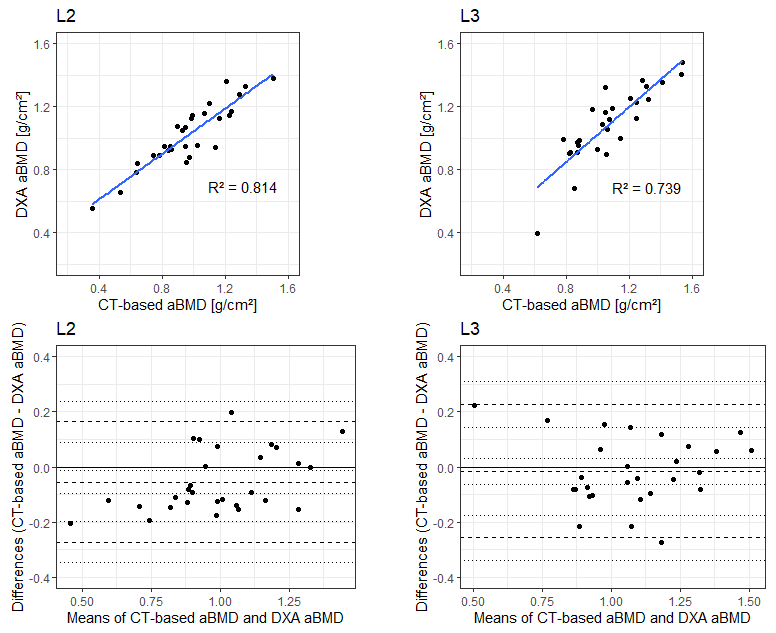

Supplement: Supplementary file 1 — (DOCX 53 kb) [file 330_2020_7655_MOESM1_ESM.docx]
